# An ecotoxicological evaluation of soil fertilized with biogas residues or mining waste

**DOI:** 10.1007/s11356-014-3927-z

**Published:** 2015-01-06

**Authors:** Krzysztof Różyło, Patryk Oleszczuk, Izabela Jośko, Piotr Kraska, Ewa Kwiecińska-Poppe, Sylwia Andruszczak

**Affiliations:** 1 Department of Agricultural Ecology, University of Life Sciences in Lublin, ul. Akademicka 13, 20-950 Lublin, Poland; 2 Department of Environmental Chemistry, Faculty of Chemistry, University of Maria Curie-Skłodowska, 3 Maria Curie-Skłodowska Square, 20-031 Lublin, Poland

**Keywords:** Biogas digestate, Mining waste, Toxicity, *Vibrio fischeri*, *Daphnia magna*, *Lepidium sativum*

## Abstract

This paper presents an ecotoxicological evaluation of soil fertilized with biogas digestate (BD) or mining waste (MS). The study was performed under pot experiment conditions. BD was added at a dose of 1.5 and 3 % to the soil. MS was applied at a dose of 10 and 20 %. Samples were collected at the beginning of the experiment and after 180 and 360 days from the start of the study. In addition, a parallel experiment with the addition of CaO was done. This was designed to eliminate the adverse effect of low soil pH on the test organisms. A battery of ecotoxicological tests was used based on tests with plants (Phytotoxkit F), microorganisms (Microtox®), and crustaceans (Daphtoxkit F). In most cases, the obtained results showed that the investigated wastes had a stimulating effect on the growth of *Lepidium sativum* roots. The highest content of BD was an exception since it inhibited the growth of *L. sativum* roots. After adding BD and MS, both the luminescence of *Vibrio fischeri* and the mortality of *Daphnia magna* was at a similar level to that in the control soil. An exception was the significant increase in the mortality of *D. magna* after 48 h for soil with 3 % BD. The tests performed after 6 and 12 months showed that, with time, the toxicity of the waste-amended soil fluctuated, but ultimately decreased in most cases compared to the control. The effect of the addition of CaO on the examined toxicity parameters was different and depended on the experimental variant.

**Electronic supplementary material:**

The online version of this article (doi:10.1007/s11356-014-3927-z) contains supplementary material, which is available to authorized users.

## Introduction

The increasing demand for agricultural products and the rising prices of fertilizers have encouraged efforts to seek new solutions to increase productivity in agriculture. In recent years, problems related to the agronomic use of different types of waste have formed one of the directions of research. Positive results have been obtained from the use of sewage sludge for environmental purposes. Due to their high content of organic matter and nutrients, sewage sludges are an interesting material for the fertilization and restoration of degraded soils (Epstein 2002). However, the use of sewage sludges involves a high risk of soil and plant contamination with heavy metals and toxic compounds. Therefore, new solutions are being sought.

A relatively new material that could be an interesting source of improvement in soil properties is the residue produced during methanogenesis in a biogas plant (biogas digestate). This material is characterized by a high content of organic matter and other nutrients, but at the same time, it does not have such a high load of contaminants. An increase in the number of biogas plants has been observed in European Union countries in recent years. In 2011 in Europe, 35922.2 GWh of electricity was produced from biogas. It is estimated that this level will double by 2020 (63972 GWh according to National Renewable Energy Action Plants, NREAP). This increase in the number of biogas plants will also entail increased amounts of biogas digestate (BD) produced during these processes. The most desirable and probable direction of BD utilization will be to use it in agriculture for conditioning soil.

In spite of the beneficial properties of BD, there is a need for a comprehensive evaluation of its properties, which would exclude its potentially negative environmental effects. To prevent any irreversible damage to the environment, these materials need to be thoroughly tested, and first and foremost, their effect on the surrounding environment must be assessed. Existing research shows that BD improves the physical and chemical properties of soil, increases crop yields, and reduces the nitrate content in plants compared to mineral fertilization (Beni et al. 2012; Lopedota et al. 2013). BD also increases the content of organic C in the soil and, at the same time, reduces the rate of its transformation in comparison to non-digested input materials (Chen et al. 2012; Lopedota et al. 2013). Moreover, BD contains more mineral nitrogen than is found in organic biomass. Additionally, ammonium nitrogen (NH_4_^+^/NH_3_) found in BD is readily available to plants. When in this form, unused nitrogen is quickly released from the soil, and thus, it can be dangerous to the environment and organisms. Efficient fertilization with biogas digestate should consider its properties, and this means that it should be applied in small split doses adjusted to crop and soil requirements (Köster et al. 2011; Alburquerque et al. 2012; Lopedota et al. 2013).

Clay minerals generated as mining waste (MS) are another material which can also be used to increase the agronomic productivity of soils. They have a relatively high capacity to sorb organic and inorganic compounds (Koutsopoulou et al. 2010). Therefore, clay minerals can be used to improve the physico-chemical parameters of light soils as a result of such characteristics.

The incorporation of fertilizer materials, including wastes, into the soil is usually an irreversible process. A change in the chemical composition and physical characteristics of the soil after the inclusion of foreign material, particularly waste material, may disturb the ion balance of the soil and affect living organisms in an unpredictable way (Epstein 2002). It is extremely important to design tests to identify and exclude these undesired effects. Chemical assays that investigate the physico-chemical properties and presence of organic and inorganic contaminants are traditionally used in the assessment of the usefulness of various kinds of waste materials. Nevertheless, such investigations do not give a complete answer concerning the response of the soil environment to the presence of waste. In addition to chemical assays, ecotoxicological tests are an interesting approach. In recent years, biological assessment has attracted increasing interest. This is attributable to the possibility of a comprehensive assessment of the effect associated with the positive or negative impact of waste on the environment. Moreover, bioassessment is a valuable tool not only to detect the presence of hazardous chemicals in the environment but also to evaluate, at the same time, the effects of mixtures, with possible synergistic, additive or antagonistic effects, as well as to demonstrate the bioavailability of contaminants to different species (Oleszczuk 2008). In ecotoxicological tests, it is particularly important to determine the effect of both the aqueous fraction and the solid phase (Domene et al. 2008; Oleszczuk et al. 2012). Tests using *Vibrio fischeri* (Microtox®) bacteria and crustaceans, *Daphnia magna*, are the most frequently used tests for the liquid phase (leachates). Solid-phase testing primarily uses tests with plants (Fuentes et al. 2006; Domene et al. 2008; Oleszczuk 2010) or other organisms, e.g., invertebrates: the springtail *Folsomia candida* (Domene et al. 2010) and the earthworm species *Aporrectodea caliginosa* and *Eisenia fetida* (Hale et al. 2013). The use of different tests (for liquids and solids) enhances the risk assessment towards different organisms.

Despite the fact that BD and MS are increasingly commonly used for soil amendments, unfortunately, in the literature, there is an absence of ecotoxicological studies on these materials. The incorporation of these waste materials into the soil may involve the risk of irreversible harmful changes. Additionally, efforts should be made to identify the doses that will produce the desired fertilization effects, at the same time ensuring the safety of agro-ecosystems.

This paper presents evaluation of the ecotoxicological risk in relation to changes in the chemical properties of soil after incorporation into the soil of two different types of waste (biogas residue-BR or mining waste-MS).

## Materials and methods

### Characteristics of waste materials

Biogas digestate (BD) was collected from a biogas plant (Wikana Bioenergia Sp. z o.o., Poland). This material is a mixture of water and digested organic matter. Maize silage is used as the main substrate for energy production. The dry matter content in unprocessed BD used in the study was 8 %.

The source of clay minerals was mining waste (MS) originating from Carboniferous roof rock, bottom rock, or interlayers of the exploited coal seams in a coal mine belonging to the coal company “Bogdanka” SA (51° 32′ 33″ N 22° 99′ 99″ E, Poland). In petrographic terms, it is a mixture of mainly clays and mudstones that weather quickly. These minerals complement organic matter concentrations. The mineral composition of this waste primarily consists of silica (SiO_2_, 47 %) and aluminum oxide (Al_2_O_3_, 22 %). The physico-chemical properties of the tested waste materials used in the experiment are shown in Table 1.

**Table 1:** Chemical properties of biogas digestate (BD), mining waste (MS) and soil (podzolic soil - PS) used in the experiment

Properties	PS	BD (dry weight)	MS (dry weight)
pH (in 1 M KCl)	4.4 ± 0.23	9.9 ± 0.47	7.8 ± 0.31
TOC (g kg^−1^)	9.5 ± 0.8	633.0 ± 1.9	281.2 ± 3.2
TN (g kg^−1^)	0.4 ± 0.1	28.8 ± 0.1	3.6 ± 0.2
C/N	23.8	22.0	77.4
E (mS/cm)	1.20 ± 0.17	3.70 ± 0.25	0.84 ± 0.19
P (mg kg^−1^)	49.4 ± 4.8	5580.6 ± 29.4	14.8 ± 27.1
K (mg kg^−1^)	45.1 ± 3.5	26906.9 ± 39.8	333.8 ± 11.9
Mg (mg kg^−1^)	10.7 ± 0.7	4420.4 ± 30.4	139.8 ± 5.3
Fe (mg kg^−1^)	393.6 ± 12.9	1445.4 ± 19.6	4200.8 ± 24.3
Ca (mg l^−1^)	222.0 ± 18.5	311.6 ± 27.3	761.0 ± 37.1
S−SO_4_ (mg kg^−1^)	7.8 ± 0.8	225.1 ± 2.3	132.7 ± 1.5
B (mg kg^−1^)	0.5 ± 0.04	23.4 ± 0.15	10.1 ± 0.09
Mn (mg kg^−1^)	61.4 ± 4.5	246.1 ± 6.9	96.6 ± 5.1
Cu (mg kg^−1^)	0.5 ± 0.04	14.2 ± 0.12	14.6 ± 0.16
Zn (mg kg^−1^)	2.5 ± 0.2	145.1 ± 0.4	24.4 ± 0.7
Al (mg kg^−1^)	4505.1 ± 52.5	512.7 ± 45.2	20,870.1 ± 87.3
Na (mg kg^−1^)	603.3 ± 29.4	2900.2 ± 38.9	1450.3 ± 40.6

The values are mean of three analyses. ± means standard deviation (*n* = 3)

### Pot experiment

At the same time, two parallel experiments were carried out. Both of the experiments were carried out as a pot experiment. In the first experiment, plastic pots (16 L) were filled with podzolic soil. Next, biogas digestate (BD) or mining waste (MS) was added. BD was added at a rate of 1.5 and 3 %, which corresponded to 40 and 80 m^3^/ha of liquid digestate. MS was applied at a rate of 10 and 20 %, which corresponded to 300 and 600 t/ha. The basis for determining the above rates was the macronutrient content in the investigated materials (Table 1). The content of the major fertilizer nutrients (NPK) in the dry weight of digestate is many times higher than in the dry weight of clay rock. In order to avoid over-fertilization with N (according to Polish regulations, the maximum rate of N is 170 kg N/ha/year) and the related increase in toxicity to soil flora and fauna, the BD rates were lower than the MS rates. In the second experiment, the experimental protocol of the first part was the same, but CaO was additionally added (0.13 %−4 t/ha) to soil. This was designed to eliminate the adverse effect of low soil pH on the test organisms. The pot experiment was carried out in three replications (three replications per pot).

The sub-samples were taken from the entire length of the pot with a stainless steel corer (2 cm in diameter). Five sub-samples from each pot were taken and were mixed to obtain a representative sample. Samples were collected at the beginning of the experiment as well as after 180 and 360 days from the start of the study. After homogenization, the soil samples were air-dried and passed through a 2-mm sieve. Then, the samples were kept in glass jars (previously cleaned by rinsing with acetonitrile) and stored in a laboratory freezer (−4 °C) before chemical and ecotoxicological (Phytotoxkit F, Daphtoxkit F and Microtox®) analysis.

### Toxicity tests

To evaluate the effect of waste-amended soil on bacteria and crustaceans, elutriates from the soils were tested (for Daphtoxkit F and Microtox®). Elutriates were obtained according to the EN 12457-2 protocol (EC 2002). The soils were mixed with de-ionized water in a single-stage batch test performed at a liquid-to-solid (L/S) ratio of 100 g/L. The glass bottles were shaken for 24 h in a roller-rotating device at 10 rpm. The extracts were filtered through a filter with a porosity of 0.45 μm.

The Microtox® Toxicity Test was used to evaluate the inhibition of the luminescence in the marine bacteria *V. fischeri* according to the test protocol (SDI 1992). The tests were carried out using a MicrotoxM500 analyzer. The light output of the luminescent bacteria from the soil elutriates was compared with the light output of a blank control sample. Luminescence inhibition of the extract was assessed for 15 min of exposure carrying out the “81.9 % basic test protocol” (MicrotoxOmni software was used). "81.9 Basic test protocol" is screening test. This procedure is used usually for "not-very-toxic" samples. In this protocol, 1 ml of undiluted extract and 100 μL of reagent (to regulate osmotic pressure) are used as a sample (which consists of 81.9 % investigated extract).

The Daphtoxkit F™ acute test with *D. magna* makes use of neonates hatched from dormant eggs (ephippia) to determine the inhibition of mortality after 24 h. Tests were performed according to the standard operational procedure manual of the Daphtoxkit F™ (Daphtoxkit 1996), which follows the OECD Guideline 202.

Phytotoxicity of soil solid samples was assessed by the commercial solid toxicity bioassay, Phytotoxkit F™ (Phytotoxkit 2004), with *Lepidium sativum*. Artificial OECD soil (OECD 1984) was used as a reference soil in the present experiment. The analyses and the root length measurements were performed after 72 h (according to procedure) using the Image Tool 3.0 for Windows (UTHSCSA, San Antonio, USA). The bioassays were performed in three replicates.

### Chemical assays

All samples from pot experiments (all terms and variants) were analyzed for physico-chemical properties. The following soil properties were analyzed using standard laboratory procedures (van Reeuwijk 1992): particle size distribution by the hydrometer method, pH in 1 M KCl solution (soil to solution ratio of 1:2.5) potentiometrically, and total nitrogen was determined by Kjeldahl’s method without the application of Dewarda’s alloy (Cu–Al–Zn alloy reducer of nitrites and nitrates).

The total organic carbon content was determined by the gravimetric method. The soil was dried at a temperature of 103 °C to constant weight and subsequently incinerated at 550 °C, and the weight loss was measured (van Reeuwijk 1992). The contents of available forms of phosphorus, potassium, and magnesium were determined by the method of Egner et al. (1960). Available forms of manganese and other compounds (B, Mn, Cu, Zn, Fe, Al, and Na) were determined in 1 M HCl by atomic absorption spectrometry (Welz 1999).

### Statistical analysis

The differences between each treatment and the control as well as between treatments were evaluated (Statistica 5.0; StatSoft, Tulsa, OK, USA) using a two-way analysis of variance (ANOVA and Tukey’s post hoc test). *P* values <0.05 were regarded as significant. The relationships between the ecotoxicological parameters, physico-chemical properties, and contaminants were determined by correlation analysis with Statistica 5.0. Significance was set at *P* < 0.05.

## Results

### Changes in the chemical properties of both the control and BD/MS-amended soils

Application of BD or MS slightly increased the pH values of the soil (Table 2). It was observed that MS increased the soil pH more than BD. Increases in pH after addition of BD and MS were associated with the initial pH and with the doses of the input material. However, in both cases, this increase was not significant.

**Table 2:** Chemical properties of the tested soil (without CaO) with the addition of biogas digestate (BD) and mining waste (MS) directly after their application to soil

Properties	0	1.5 % BD	3 % BD	10 % MS	20 % MS	HSD_0.05_
pH (in 1 M KCl)	4.38 ± 0.24	4.68 ± 0.27	4.92 ± 0.32	5.20 ± 0.29	5.45 ± 0.37	0.41
TOC (g kg^−1^)	9.26 ± 0.75	11.56 ± 0.61	12.67 ± 0.93	17.61 ± 0.70	21.61 ± 0.89	2.01
TN (g kg^−1^)	0.35 ± 0.01	0.47 ± 0.02	0.56 ± 0.03	0.66 ± 0.02	0.70 ± 0.03	0.03
C/N	26.5	24.6	22.6	26.7	30.9	
P (mg kg^−1^)	51.5 ± 4.0	56.3 ± 4.3	73.7 ± 4.8	49.3 ± 5.5	53.7 ± 4.7	6.2
K (mg kg^−1^)	52.3 ± 3.9	97.1 ± 5.7	224.2 ± 7.8	83.0 ± 4.9	80.5 ± 5.3	7.5
Mg (mg kg^−1^)	10.2 ± 0.8	14.9 ± 0.9	26.5 ± 2.4	31.7 ± 3.2	34.0 ± 3.8	4.6
Fe (mg kg^−1^)	395 ± 26	394 ± 30	410 ± 32	500 ± 33	538 ± 37	36
Ca (mg l^−1^)	180 ± 11	218 ± 10	273 ± 14	270 ± 19	278 ± 17	15
S−SO_4_ (mg kg^−1^)	3.6 ± 0.4	4.4 ± 0.5	14.5 ± 1.2	22.3 ± 1.8	32.3 ± 1.9	1.5
B (mg kg^−1^)	0.30 ± 0.04	0.73 ± 0.09	1.08 ± 0.08	1.04 ± 0.12	1.23 ± 0.09	0.14
Mn (mg kg^−1^)	64.9 ± 4.1	66.5 ± 4.9	76.9 ± 6.7	94.5 ± 7.8	102.2 ± 7.5	7.3
Cu (mg kg^−1^)	0.49 ± 0.04	0.54 ± 0.08	0.68 ± 0.06	1.10 ± 0.15	1.51 ± 0.16	0.13
Zn (mg kg^−1^)	2.02 ± 0.17	2.14 ± 0.16	2.28 ± 0.19	2.75 ± 0.34	3.40 ± 0.33	0.21
Al (mg kg^−1^)	4505 ± 67	3649 ± 74	3789 ± 69	4802 ± 61	4740 ± 70	154
Na (mg kg^−1^)	653 ± 34	914 ± 78	891 ± 92	697 ± 41	702 ± 55	48

The values are mean of three analysis. ±means standard deviation (*n* = 3)

*HSD*_*0.05*_ the honestly significant difference

The addition of MS to the soil significantly increased the C content by 90 % for the 10 % dose and by 130 % for the 20 % dose relative to the control soil. Significant increase in N content by 90 and 130 % respectively was also observed. For BD, the increase in C content was not so high like for MS and was at the level of 25 and 37 % for the doses of 1.5 and 3 %, respectively. However, the N content was found to increase greatly (by 34 and 60 %, respectively). This caused a decrease in the C/N ratio compared to the control soil (Table 2). It should be emphasized that the C/N ratio in BD and MS-amended soil was still high (>22). According to Alexander (1977), a C/N ratio above 20 may cause N immobilization.

The addition of BD to the soil caused a significant increase in K and P in the amended soil (by several orders of magnitude in some cases), which was attributable to the high content of these elements in the input material (Table 1). For MS, only an increase in K content was observed (Table 2). Because of the low content of P in MS, the content of P after the addition of MS did not change significantly (Table 1). The content of the available forms of Mg and Fe also increased significantly after the addition of both MS and BD. The Mg concentration in BD was significantly higher than in MS, but a higher content of Mg in the test soil was found after the addition of MS. The application of both MS and BD to the soil resulted in an increase in the content of the available forms of other macro- and micronutrients (Table 2). It should, however, be stressed that after adding BD to the soil, a decrease in the amount of the available Al was observed, which is probably related directly to the increase in pH of the waste-amended soils.

The chemical analyses performed after 6 and 12 months did not show significant directional changes in the chemical properties of the soil in relation to the analyses conducted immediately after BD and MS addition (Tables 3 and 4). The determined coefficients of variation did not exceed the level of 10 %.

**Table 3:** Chemical properties of the tested soil (without CaO) with the addition of biogas digestate (BD) and mining waste (MS) 6 months after their application to soil

Properties	0	1.5 % BD	3 % BD	10 % MS	20 % MS	HSD_0.05_
pH (in 1 M KCl)	4.32 ± 0.28	4.52 ± 0.30	4.78 ± 0.27	4.83 ± 0.32	4.97 ± 0.33	0.35
TOC (g kg^−1^)	9.75 ± 1.18	10.90 ± 1.02	12.13 ± 1.08	16.09 ± 1.22	19.39 ± 1.30	1.56
TN (g kg^−1^)	0.34 ± 0.02	0.43 ± 0.01	0.55 ± 0.02	0.49 ± 0.02	0.64 ± 0.02	0.02
C/N	28.7	25.3	22.1	32.8	30.3	
P (mg kg^−1^)	48.9 ± 3.9	55.8 ± 3.6	68.1 ± 4.0	44.5 ± 4.1	48.0 ± 4.2	5.7
K (mg kg^−1^)	42.3 ± 3.8	95.5 ± 5.0	206.7 ± 6.4	51.5 ± 5.1	57.3 ± 4.6	6.9
Mg (mg kg^−1^)	12.3 ± 0.8	13.9 ± 0.8	24.5 ± 1.2	30.2 ± 1.9	41.4 ± 1.7	4.1
Fe (mg kg^−1^)	401 ± 37	386 ± 28	381 ± 25	428 ± 34	476 ± 39	33
Ca (mg l^−1^)	270 ± 32	235 ± 19	330 ± 26	247 ± 37	520 ± 40	36
S−SO_4_ (mg kg^−1^)	9.5 ± 1.0	11.3 ± 0.9	10.9 ± 0.8	28.9 ± 2.1	65.2 ± 2.7	2.5
B (mg kg^−1^)	0.49 ± 0.06	0.83 ± 0.05	1.52 ± 0.11	0.83 ± 0.09	1.21 ± 0.10	0.12
Mn (mg kg^−1^)	65.0 ± 3.4	62.8 ± 4.8	74.4 ± 4.5	69.4 ± 6.8	82.6 ± 7.1	6.9
Cu (mg kg^−1^)	0.52 ± 0.05	0.65 ± 0.07	0.46 ± 0.14	1.39 ± 0.12	1.64 ± 0.17	0.14
Zn (mg kg^−1^)	2.67 ± 0.15	2.81 ± 0.17	3.27 ± 0.16	3.52 ± 0.31	4.18 ± 0.35	0.28
Al (mg kg^−1^)	4370 ± 52	3834 ± 79	3651 ± 67	4586 ± 110	5294 ± 125	163
Na (mg kg^−1^)	702 ± 45	892 ± 68	825 ± 79	706 ± 53	698 ± 57	51

The values are mean of three analysis. ± means standard deviation (*n* = 3)

*HSD*_*0.05*_ the honestly significant difference

**Table 4:** Chemical properties of the tested soil (without CaO) with the addition of biogas digestate (BD) and mining waste (MS) 12 months after their application to soil

Properties	0	1.5 % BD	3 % BD	10 % MS	20 % MS	HSD_0.05_
pH (in 1 M KCl)	4.49 ± 0.26	4.76 ± 0.24	5.08 ± 0.29	5.05 ± 0.25	5.19 ± 0.27	0.38
TOC (g kg^−1^)	9.19 ± 0.89	11.72 ± 1.04	12.52 ± 1.09	17.81 ± 0.95	21.27 ± 1.21	1.20
TN (g kg^−1^)	0.32 ± 0.01	0.41 ± 0.01	0.46 ± 0.02	0.43 ± 0.01	0.51 ± 0.02	0.02
C/N	28.7	28.6	27.2	41.4	41.7	
P (mg kg^−1^)	45.8 ± 3.2	63.3 ± 3.5	77.7 ± 3.9	51.9 ± 3.3	55.8 ± 3.8	5.4
K (mg kg^−1^)	44.8 ± 3.9	91.3 ± 5.5	178.5 ± 6.9	66.4 ± 4.3	68.9 ± 4.1	6.6
Mg (mg kg^−1^)	9.79 ± 0.7	15.1 ± 1.5	24.3 ± 1.3	33.2 ± 1.8	39.1 ± 1.6	3.5
Fe (mg kg^−1^)	398 ± 19	390 ± 29	395 ± 25	464 ± 26	507 ± 31	28
Ca (mg l^−1^)	245 ± 17	261 ± 20	321 ± 24	278 ± 29	393 ± 22	25
S−SO_4_ (mg kg^−1^)	6.2 ± 0.4	8.7 ± 0.6	13.4 ± 0.9	27.5 ± 1.8	49.7 ± 2.0	1.7
B (mg kg^−1^)	0.35 ± 0.04	0.87 ± 0.09	1.29 ± 0.07	1.08 ± 0.08	1.29 ± 0.09	0.10
Mn (mg kg^−1^)	65.3 ± 4.7	64.2 ± 5.2	75.5 ± 3.9	81.3 ± 6.4	92.4 ± 7.0	6.5
Cu (mg kg^−1^)	0.48 ± 0.04	0.55 ± 0.04	0.67 ± 0.06	1.13 ± 0.10	1.57 ± 0.13	0.10
Zn (mg kg^−1^)	2.41 ± 0.12	2.45 ± 0.13	2.63 ± 0.15	3.01 ± 0.29	3.72 ± 0.26	0.19
Al (mg kg^−1^)	4520 ± 49	4017 ± 62	3875 ± 85	4862 ± 107	5140 ± 96	132
Na (mg kg^−1^)	623 ± 30	979 ± 76	742 ± 70	617 ± 39	661 ± 42	44

The values are mean of three analysis. ± means standard deviation (*n* = 3)

*HSD*_*0.05*_ the honestly significant difference

Adding CaO to the soil caused a significant increase in pH. This increase had an effect on N, and the content of which significantly increased (Table S1). This resulted in a decrease in the C/N ratio compared to the soil without CaO addition. In most of the samples analyzed, the increase in pH after the application of CaO also caused a significant increase in the content of the available forms of macro- and micronutrients. An exception to this process was the K content in soil with 3 % of BD and 10 % of MS (Table S1).

### Influence of wastes on soil toxicity

**Fig. 1:**
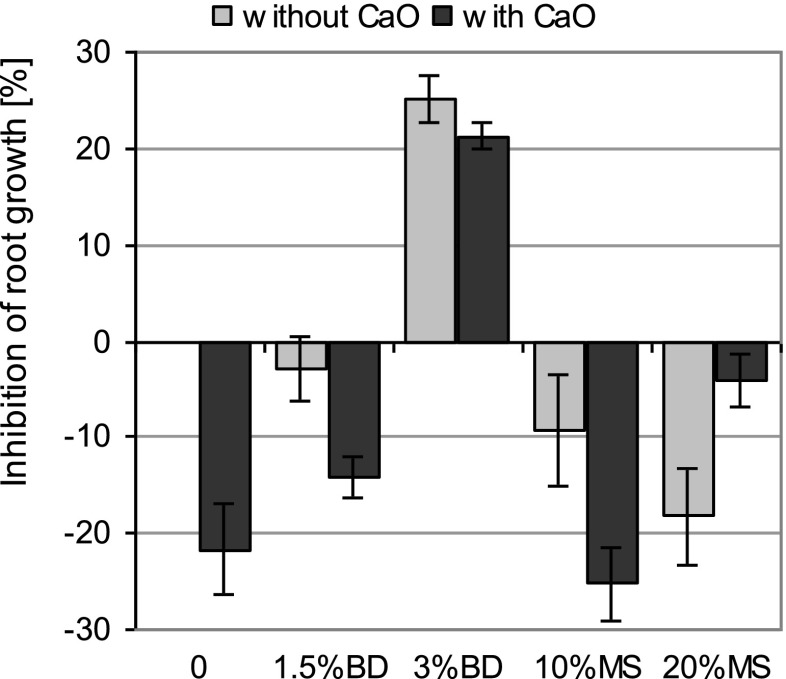
Effect of the dose of biogas digestate (*BD*) and mining waste (*MS*) on root growth inhibition of *Lepidium sativum. Error bars* represent the standard deviation of the mean (*n* = 3)

Figure 1 shows the influence of the tested materials on root growth inhibition (RGI) at the beginning of the experiment (term I). The addition of BD to the control soil at a dose of 1.5 % caused the stimulation of root growth relative to the unamended soil without CaO. The stimulation effect was more significant than the treatment with CaO. An increase in the dose of BD to 3 % resulted in a significant increase in phytotoxicity (>20 %). In this case, no significant difference was found between those soils with CaO and those without CaO. The MS-amended soil significantly stimulated root growth compared to the control soil. CaO had a contrasting effect depending on the MS dose. In the presence of the lowest dose of MS, the stimulation of root growth with CaO was stronger. A reverse trend was, however, observed for the higher MS dose. In this case, the presence of CaO contributed to a significant reduction in the stimulating effect of MS (Fig. 1).

**Fig. 2:**
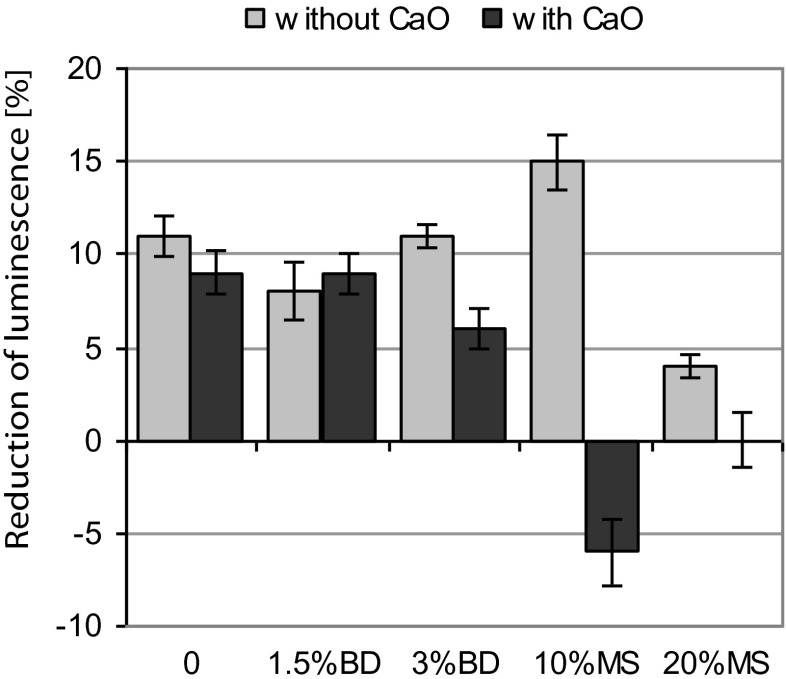
Effect of the dose of biogas digestate (*BD*) and mining waste (*MS*) on the reduction of *Vibrio fischeri* luminescence. *Error bars* represent the standard deviation of the mean (*n* = 3)

**Fig. 3:**
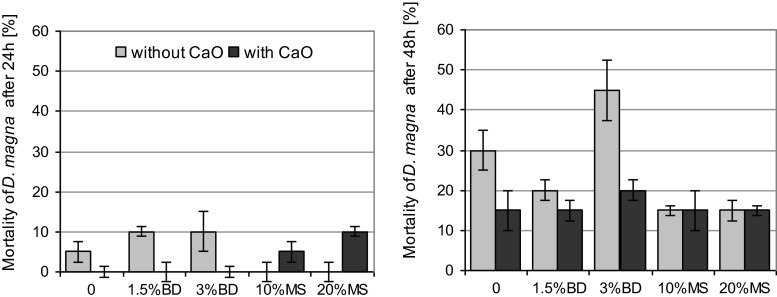
Mortality of *Daphnia magna* in biogas digestate (*BD*) and mining waste (*MS*)-amended soil after 24 and 48 h of exposure. *Error bars* represent the standard deviation of the mean (*n* = 3)

The effect of elutriates obtained from the investigated soils on the inhibition of *V. fischeri* luminescence and on the mortality of *D. magna* is presented on Figs. 2 and 3. Elutriates from the control soils caused an 11 % inhibition of *V. fischeri*. The presence of CaO did not significantly affect the result (Fig. 2). The addition of BD to the soil, at the doses of 1.5 and 3 %, did not have a significant effect on the luminescence of *V. fischeri* in relation to the control soil. A slight reduction of luminescence inhibition was only found in soil with CaO and BD addition at the rate of 3 %. On the other hand, MS had a significant effect on the luminescence of *V. fischeri*. The effect of the MS amendment was clearly dependent on both the MS dose and the presence of CaO. Soil with 10 % MS addition increased the inhibition of *V. fischeri* luminescence up to a level of 15 %, whereas an MS addition rate of 20 % reduced this inhibition to 4 %. In the presence of CaO, MS positively influenced the luminescence of *V. fischeri*, irrespective of its rate, compared to the control soil (Fig. 2).

Figure 3a, b demonstrates the effect of elutriates on *D. magna* mortality after 24 and 48 h of exposure. In the control soil, the mortality of *D. magna* was 5 and 30 % after 24 and 48 h, respectively (Fig. 3). This indicates relatively the adverse conditions for the development of *D. magna*. The application of BD, regardless of its dose, led to a twofold increase in the mortality of *D. magna* after 24 h (Fig. 3a). After 48 h, the observed effect was dependent on the dose. A lower dose of BD (1.5 %) caused a decrease in soil toxicity to *D. magna*, whereas a higher dose resulted in a significant increase in toxicity.

The addition of MS to the soil eradicated the toxic effect of the elutriates observed in the control soil (Fig. 3a). A significant reduction in toxicity was also observed after 48 h (Fig. 3b). However, it should be emphasized that the determined values are greater than the mean which illustrates the great variability of the response of *D. magna*. The presence of CaO significantly influenced the observed toxic effect both after 24 h (all the soil amendments) and 48 h (only for BD). In the case of BD, both after 24 and 48 h, the toxicity of elutriates was found to decrease in the presence of CaO, while the use of MS and CaO significantly increased the toxicity of the tested samples in relation to the control soil containing only CaO.

### Changes of ecotoxicological properties

**Fig. 4:**
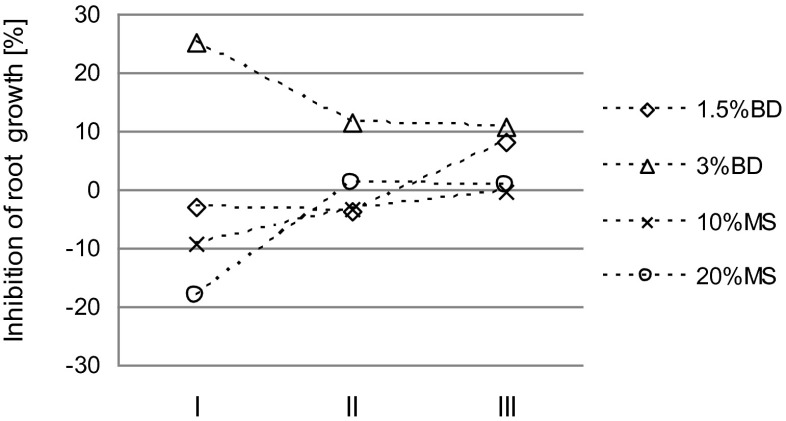
Changes in *Lepidium sativum* root growth inhibition in biogas digestate (*BD*) and mining waste (*MS*)-amended soil (*I* directly after material application, *II* after 6 months, *III* after 12 months)

Figure 4 shows the change over time of the phytotoxicity of soil amended with the tested materials. After 6 months from the beginning of the study, a significant reduction in *L. sativum* root growth inhibition (by 14 %) was found for the 3 % dose of BD compared to the beginning of the experiment. It should be underlined that this was the only experimental treatment where an increase in phytotoxicity was observed at the beginning of the research. The stimulation of root growth observed at the beginning of the study was found to decrease in the case of MS (Fig. 4). Nevertheless, the values obtained in this case were still better than those for the control soil. In soil amended with a 1.5 % rate of BD, no significant changes were found during the first 6 months in relation to the beginning of the experiment. During the period from the 6th to the 12th month, no significant changes in phytotoxicity were observed for the other experimental treatments, except for the 1.5 % dose of BD (Fig. 4). On the last assessment date, no significant differences were found between the toxicity of the control soil and the MS-amended soil. Regardless of the dose, BD showed higher toxicity to plants than the control soil and MS-amended soil.

**Fig. 5:**
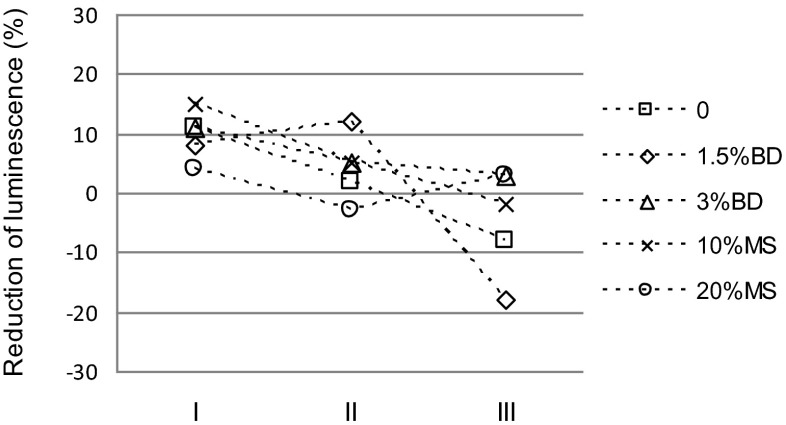
Changes in *Vibrio fischeri* luminescence reduction in elutriates from biogas digestate (*BD*) and mining waste (*MS*)-amended soil (*I* directly after material application, *II* after 6 months, *III* after 12 months)

Both in the control soil and in the soil amended with the investigated materials, a significant decrease in their toxicity to *V. fischeri* was observed (Fig. 5). The highest reduction in toxicity, in relation to the beginning of the study, was found for the 10 % dose of MS (this dose alone demonstrated significantly higher luminescence inhibition relative to the control soil at the beginning of the study), the control soil, and the 1.5 % dose of BD. For all the experimental treatments, the level of *V. fischeri* inhibition was lower than at the beginning of the study. However, this inhibition was lower than that for the control soil only in the case of the 1.5 % dose of BD.

**Fig. 6:**
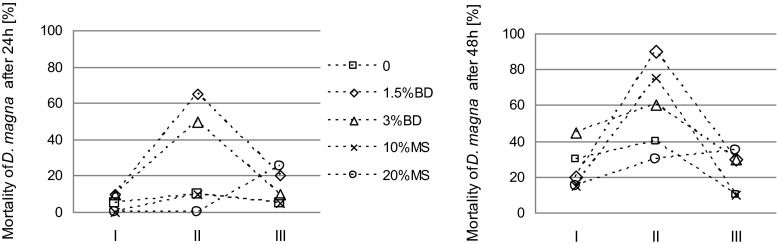
Changes in *Daphnia magna* mortality in elutriates from biogas digestate (BD) and mining waste (*MS*)-amended soil (*I* directly after material application, *II* after 6 months, *III* after 12 months)

Figure 6 presents the mortality of *D. magna*. A significant increase in *D. magna* mortality was found in all the treatments (except for the 20 % rate of MS after 24 h). A particularly high increase in *D. magna* mortality was found in soils with BD (for 24 h), as well as in soils with BD (1.5 %) and MS (10 %) for 48 h. During the next 6 months (from the 6th to the 12th month of the study), a significant reduction in the mortality of *D. magna* was observed, except for the soil with 20 % MS addition (Fig. 6). At the last assessment time, the mortality of *D. magna* was higher in the amended soil than in the control. An exception was the soil with 10 % MS addition; in the case of which, the values were similar to those obtained for the control soil.

## Discussion

The addition of the investigated materials (BD and MS) to the soil significantly changed its chemical and ecotoxicological properties. The effect of these soil amendments depended on the kind of material and the ecotoxicological test used. To date, no research has been conducted on the toxicity of BD- or MS-amended soils. Currently, the results only refer to biosolids, which have been commonly studied and assessed (Fuentes et al. 2006; Ramírez et al. 2008; Domene et al. 2010; Koutsopoulou et al. 2010; Oleszczuk et al. 2012; Malara and Oleszczuk 2013). Unlike biosolids, in most cases, the studied materials only had an insignificant effect on the phytotoxicity of the amended soils. Only a 3 % dose of BD caused a significant inhibition of root growth. This could have been related to over-fertilization with potassium (Schachtman and Schroeder 1994; Mäser et al. 2002) which was present in an excessive amount in BD (Table 1). The application of BD at the lower dose and of MS at both doses had a positive effect on plant growth at the beginning of the experiment. In some cases, stimulation of root growth was also observed in different research terms. This was associated with an increase in the content of nutrients and a pH rise and thus also increased availability of macro- and micronutrients (Cramer et al. 1991). BD and MS contained large amounts of organic carbon (Table 1). The presence of natural organic substances may affect the bioavailability of elements (Spurgeon and Hopkin 1996; Kim et al. 1999; Domene et al. 2008), limiting their negative effect on organisms (Domene et al. 2010). When comparing our results to other reports (Oleszczuk 2010; Oleszczuk et al. 2012), it should be stressed that skillful application of BD and MS results in a better effect on soils than the application of biosolids. For example, our studies showed a significant limitation in plant development and growth when sewage sludges were added to different soils. Likewise, Fuentes et al. (2006) and Ramírez et al. (2008) found an inhibiting influence of sewage sludges on the germination and root growth of many plant species.

The tested materials only slightly affected the toxicity in *V. fischeri*. In most cases (BD 1.5, 3 %, and MS 20 %), the positive or insignificant effect of the materials was observed in relation to the unamended soil. *V. fischeri* is frequently used as an indicator of environmental contamination with chemical compounds of different kinds. However, their use in the evaluation of biosolids is insignificant. Earlier research (Hale et al. 2013; Malara and Oleszczuk 2013) has shown that *V. fischeri* constitutes a test that is not sensitive to the toxicity of wastes or contaminated soils. Even in the case of such controversial waste as sewage sludge, this sludge is observed to have only a slight effect on the luminescence of *V. fischeri*. The sensitivity of this test is additionally reduced after sewage sludge is mixed with soil (Malara and Oleszczuk 2013).

The observed reduction in phytotoxicity (or stimulation) after adding the studied materials may be related to the positive effect that these materials have on the growth and development of organisms. It should be stressed that both BD and MS are rich in macro- and micronutrients and increase soil pH. Another factor, especially in cases when a high toxicity of the control soil was observed (e.g., *D. magna*; Fig. 3b), is that the decreased toxicity may be attributable to the “dilution” of the soil by a less harmful material. A similar phenomenon has been observed in previous research (Malara and Oleszczuk 2013), where extracts obtained from unamended soil were characterized by high toxicity to *D. magna*. After the addition of sewage sludge, a significant reduction in *D. magna* mortality was observed (Malara and Oleszczuk 2013).

The studied soil was characterized by a low pH, which may have a direct negative effect on all the organisms tested. It should, however, be emphasized that under acidic conditions, it was reported that the solubility of many pollutants increases (Wang 2009), and consequently, they can have an additional negative effect on organisms. Adding CaO to soil increases its pH (Table S1); in most cases, it substantially eliminated the negative effect on the studied organisms.

With time, the effect of the investigated materials on the tested ecotoxicological parameters changed. The decrease in toxicity (Fig. 4, 3 % BD; Fig. 5) was probably related to the reduced action of the toxic factor as a result of its degradation or being washed out. A decrease in the stimulating effect of the tested materials on *L. sativum* was also observed. This could have been caused by the decreased availability of nutrients (in some cases) or by organic carbon transformations. The most surprising changes were found in the case of *D. magna*. The observed significant increase in toxicity after 6 months could, on the one hand, be associated with the activation of contaminants which were unavailable earlier as a result of organic matter mineralization. This phenomenon has previously been observed in relation to sewage sludge (Smith et al. 2001; Oleszczuk 2006). Chen et al. (2012) observed that during a 21-day period of incubation, 6.4 % of organic carbon compounds derived from BGR (biogas residues) became mineralized, which may confirm earlier suppositions. Another possible explanation for the observed phenomenon is the formation of intermediate compounds. The issue of the transformations of various compounds during the application of biogas residues to amend soils is still poorly researched, and hence, it is difficult to indicate the factors responsible for the observed increase in toxicity.

To identify potential factors responsible for the observed toxicity, a correlation analysis was performed. We decided not to discuss values showing a stimulating effect on tested organisms. Significant correlations that could show a potentially toxic effect of the individual components of the tested materials on *L. sativum* relate to the negative correlation of Na and P content with root length (BD 1.5 %/Table S2), pH and S-SO_4_ content with root length (BD3 %/Table S3), and Mg content with root length (MS10 %/Table S4). The negative effect of the experimental factors on *D. magna* and *V. fischeri* relate to the positive correlations (an increase in the intensity of the factor increased mortality/inhibition of luminescence). Significant correlations occurred between the mortality of *D. magna* after 24 h and the increase in the C/N ratio and the content of P and Ca (1.5 %BD/Table S2), whereas after 48 h, such correlations existed between the mortality and C/N and P (1.5 %BD/Table S2), Al (3 %BD/Table S3), Mg, Ca, S−SO_4_, Cu, Al, and Zn (20 %MS/Table S5). The inhibition of *V. fischeri* luminescence was correlated both with the increase in pH and C content (1.5 %BD/Table S2) and with the increase in the C/N ratio, as well as in the content of Fe and Mn (3 %BD/Table S3). After adding 10 % of MS (Table S4), significant correlations were found between the reduced luminescence and pH as well as the contents of N, K, Fe, and Mn, whereas at the higher rate of MS (Table S5), the inhibition of luminescence was related to the Na content.

An excessive concentration of NO_3_^−^ and K^+^, followed by Na^+^, Cl^−^, and SO_4_^2+^, can cause soil salinity and related toxicity to organisms. In terms of toxicity to plants, a safe limit is considered to be 130 mM NaCl or KCl up to 130 mM in culture solutions or high Na^+^ (2 mg g^−1^ FW) or K^+^ (4 mg g^−1^ FW) in the root tissue provided that [Ca^2+^] > 2 mM in the rooting medium (Kinraide 1999; Khan and Weber 2006). Most crop plants are more sensitive to salinity during vegetative growth and development (the effect of salt accumulation in the leaves) than at the germination stage. The inhibition of plant germination is a result of the osmotic effect and is similar to the initial response to water stress (Jenks et al. 2007). Ca^2+^ partially mitigates the inhibition of growth. The Ca^2+^ effect appears to be related to the maintenance of plasma membrane selectivity for K over Na (Kinraide et al. 2004). This author also draws attention to the interactions of salinity with other stress factors, among others is boron toxicity, but the mechanisms of these interactions are still poorly understood. This is confirmed by the studies of Alburquerque et al. (2012) and Lopedota et al. (2013) who suggest that heavy metals (primarily Cu and Zn) and salinity, and thus an increase in phytotoxicity are a limitation of BD use in agriculture. The present study did not find a significant increase in Cu content in the soil after adding BD, but an increased content of Zn was confirmed, whereas the MS amendment increased the soil concentration of Cu and Zn (Table 2). However, this was not translated into an increase of phytotoxicity to *L. sativum*. The BD-amended soil contained significantly less Cu and Zn than the soil with MS, but in spite of this, it was characterized by higher toxicity (Fig. 1). This demonstrates that the most probable reason for toxicity to the test organisms was the high content of Na and other elements (K, S−SO_4_, and N) and this resulted in increased soil salinity (Mäser et al. 2002).

Tsiridis et al. (2006) reported that the toxicity of Cu to *V. fischeri* was higher than that of Zn. In their research, Cu toxicity decreased after adding humic acids, while Zn toxicity remained at the same level. This is associated with the significantly higher effectiveness of Cu to form complexes than is the case with Zn. As a result of the formation of Cu complexes with humic acids, a reduction in the toxicity to *V. fischeri* was observed, and this was determined by the activity of the free ion (Tsiridis et al. 2006). These results are in agreement with the results of Kim et al. (1999) who reported that the formation of Cu complexes with organic ligands leads to decreased toxic activity of this element to test organisms. In our results, a significant increase in Cu content after adding MS was also accompanied by a significantly higher increase in TOC and pH than what occurred after adding BD (Table 2). This resulted in Cu immobilization and the absence of an increase in toxicity. As reported by Domene et al. (2010), higher organic carbon contents and, at the same time, the lower toxicity of sludge are probably related to the higher sorption capacity of the soil. The described relationships could be an explanation of the reduced toxicity found at successive observation times during the present study. With the passage of time, organic matter incorporated together with the waste materials reduced the toxic effect of the elements on *L. sativum* and *V. fischeri* after adding both BD and MS (Figs. 1 and 2).

## Conclusion

Our results showed that the studied materials could be a good material to improve soil properties. In most cases, the addition of the studied materials to the soils had a positive, though insignificant, effect on the tested organisms in relation to the control soil. In cases where a negative impact of tested materials was observed, this decreased gradually. Additionally, it should be emphasized that after 12 months of the experiment, the level of toxicity in BD or MS-amended soil was similar or even lower than that in unamended soil.

## Electronic supplementary material

Below is the link to the electronic supplementary material.

ESM 1 (DOCX 46.4 kb)
