# Supplementary material for: An ecotoxicological evaluation of soil fertilized with biogas residues or mining waste
Source: Environ Sci Pollut Res Int. 2015 Jan 6;22(10):7833–42. doi: 10.1007/s11356-014-3927-z (PMC4432083; doi:10.1007/s11356-014-3927-z)
Supplement: Supplementary file 1 — (DOCX 46.4 kb) [file 11356_2014_3927_MOESM1_ESM.docx]

SUPPLEMENTARY INFORMATION

**AN ECOTOXICOLOGICAL EVALUATION OF SOIL FERTILIZED WITH BIOGAS RESIDUES OR MINING WASTE**

Krzysztof Różyło^1^, Patryk Oleszczuk^2*^, Izabela Jośko^2^, Piotr Kraska^1^, Ewa Kwiecińska-Poppe^1^, Sylwia Andruszczak^1^

*^1^Department of Agricultural Ecology, University of Life Sciences in Lublin, ul. Akademicka 13, 20-950 Lublin, Poland*

*^2^Department of Environmental Chemistry, Faculty of Chemistry, University of Maria Curie-Skłodowska, 3 Maria Curie-Skłodowska Square, 20-031 Lublin, Poland.*

**corresponding author*: Patryk Oleszczuk, patryk.oleszczuk@poczta.umcs.lublin.pl; phone: +48 81 5375515; fax: +48 81 5375565.

Journal: Environmental Science and Pollution Research

Number of pages: 6 (including this page)

Number of tables: 5

**Table S1.** Chemical properties of the tested soil with the addition of biogas digestate (BD) and mining waste (MS) - mean for 3 assessment times

| **Properties** | **0** | | **0+1.5%BD** | | **0+3%BD** | | **0+10%MS** | | **0+20%MS** | |
| --- | --- | --- | --- | --- | --- | --- | --- | --- | --- | --- |
|  | A* | B | A | B | A | B | A | B | A | B |
| **pH** (in 1MKCl) | 4.4 | 6.9 | 4.7 | 7.6 | 4.9 | 7.6 | 5.0 | 7.6 | 5.2 | 7.8 |
| **C** (g·kg^-1^) | 9.40 | 9.40 | 11.39 | 11.27 | 12.44 | 12.03 | 17.17 | 19.17 | 20.76 | 20.97 |
| **N** (g·kg^-1^) | 0.34 | 0.43 | 0.44 | 0.51 | 0.52 | 0.60 | 0.53 | 0.56 | 0.62 | 0.54 |
| **C/N** | 28.0 | 22.0 | 26.2 | 22.1 | 24.0 | 20.1 | 33.5 | 34.2 | 34.0 | 38.6 |
| **P** (mg·kg^-1^) | 48.73 | 55.54 | 58.47 | 80.58 | 73.17 | 84.08 | 48.57 | 62.26 | 52.50 | 61.39 |
| **K** (mg·kg^-1^) | 46.47 | 46.49 | 94.63 | 125.94 | 203.13 | 178.49 | 66.97 | 60.36 | 68.90 | 75.30 |
| **Mg** (mg·kg^-1^) | 10.93 | 10.7 | 14.63 | 19.7 | 25.10 | 20.0 | 31.70 | 30.3 | 38.17 | 43.0 |
| **Fe** (mg·kg^-1^) | 398.0 | 401 | 390.0 | 412 | 395.3 | 413 | 464.0 | 522 | 507.0 | 505 |
| **Ca** (mg·l^-1^) | 231.7 | 1045.3 | 238.0 | 1567.7 | 308.0 | 1846.3 | 265.7 | 1422.1 | 397.8 | 1316.3 |
| **S-SO_4_** (mg·kg^-1^) | 6.43 | 11.8 | 8.13 | 15.7 | 12.93 | 14.1 | 26.23 | 58.1 | 49.07 | 126.2 |
| **B** (mg·kg^-1^) | 0.38 | 0.58 | 0.81 | 1.43 | 1.30 | 1.40 | 0.98 | 1.25 | 1.24 | 1.52 |
| **Mn** (mg·kg^-1^) | 65.07 | 63.6 | 64.50 | 73.0 | 75.60 | 77.3 | 81.73 | 97.1 | 92.40 | 96.7 |
| **Cu** (mg·kg^-1^) | 0.50 | 0.53 | 0.58 | 0.59 | 0.60 | 0.62 | 1.21 | 1.38 | 1.57 | 1.68 |
| **Zn** (mg·kg^-1^) | 2.37 | 2.21 | 2.47 | 2.70 | 2.73 | 2.83 | 3.09 | 3.43 | 3.77 | 3.82 |
| **Al** (mg·kg^-1^) | 4465 | 4654 | 3833 | 3921 | 3771 | 3850 | 4750 | 4861 | 5058 | 5719 |
| **Na** (mg·kg^-1^) | 659.2 | 629 | 928.7 | 803 | 819.33 | 965 | 673.33 | 643 | 687.0 | 690 |

* - A - without CaO; B - with Ca

**Table S2.** Relationships between the physico-chemical properties and ecotoxicological parameters in the soil with 1.5% dose of biogas digestate (BD)

| Properties | V. fisherii (Microtox®) | *Daphnia magna* 24h (Daphtoxkit F) | *Daphnia magna* 48h (Daphtoxkit F) | *Lepidium sativum* (Phytotoxkit F) |
| --- | --- | --- | --- | --- |
| pH | **1.00** | 0.50 | 0.53 | -0.80 |
| C | **1.00** | 0.45 | 0.48 | -0.76 |
| N | 0.07 | -0.86 | -0.84 | 0.59 |
| C/N | 0.46 | **1.00** | **1.00** | -0.92 |
| P | 0.65 | **0.97** | **0.98** | **-0.99** |
| K | 0.93 | 0.08 | 0.12 | -0.46 |
| Mg | **1.00** | 0.49 | 0.52 | -0.79 |
| Fe | 0.80 | -0.17 | -0.13 | -0.23 |
| Ca | 0.24 | **0.97** | 0.96 | -0.81 |
| S-SO_4_ | -0.71 | 0.31 | 0.27 | 0.09 |
| B | -0.12 | 0.83 | 0.81 | -0.54 |
| Mn | 0.71 | -0.31 | -0.27 | -0.09 |
| Cu | **-0.98** | -0.27 | -0.30 | 0.62 |
| Zn | -0.83 | 0.13 | 0.09 | 0.27 |
| Al | 0.12 | 0.94 | 0.92 | -0.73 |
| Na | 0.78 | 0.91 | 0.93 | **-1.00** |

in bold – statistical significant values (P≤0.05); in red – values suggesting potential toxicity (discussed in the text)

**Table S3.** Relationships between the physico-chemical properties and ecotoxicological parameters in the soil with 3% dose of biogas digestate (BD)

| Properties | V. fisherii (Microtox®) | *Daphnia magna* 24h (Daphtoxkit F) | *Daphnia magna* 48h (Daphtoxkit F) | *Lepidium sativum* (Phytotoxkit F) |
| --- | --- | --- | --- | --- |
| pH | 0.85 | 0.28 | 0.72 | **-0.99** |
| C | 0.96 | 0.00 | 0.50 | -0.91 |
| N | **-0.99** | 0.42 | -0.09 | 0.66 |
| C/N | **0.99** | -0.37 | 0.14 | -0.69 |
| P | 0.93 | 0.10 | 0.58 | -0.95 |
| K | 0.62 | -0.93 | -0.61 | 0.03 |
| Mg | 0.95 | -0.57 | -0.08 | -0.52 |
| Fe | **0.97** | -0.02 | 0.48 | -0.91 |
| Ca | **-1.00** | 0.37 | -0.15 | 0.70 |
| S-SO_4_ | 0.88 | 0.22 | 0.68 | **-0.98** |
| B | -0.95 | -0.03 | -0.52 | 0.92 |
| Mn | **0.98** | -0.07 | 0.44 | -0.88 |
| Cu | 0.72 | 0.46 | 0.85 | **1.00** |
| Zn | -0.90 | -0.17 | -0.64 | **0.97** |
| Al | 0.37 | 0.79 | **0.99** | -0.88 |
| Na | 0.67 | -0.90 | -0.56 | -0.04 |

in bold – statistical significant values (P≤0.05); in red – values suggesting potential toxicity (discussed in the text)

**Table S4.** Relationships between the physico-chemical properties and ecotoxicological parameters in the soil with 10% dose of mining waste (MS)

| Properties | V. fisherii (Microtox®) | *Daphnia magna* 24h (Daphtoxkit F) | *Daphnia magna* 48h (Daphtoxkit F) | *Lepidium sativum* (Phytotoxkit F) |
| --- | --- | --- | --- | --- |
| pH | **1.00** | -0.51 | 0.05 | -0.26 |
| C | 0.78 | 0.05 | 0.60 | -0.75 |
| N | **0.99** | -0.71 | -0.20 | -0.01 |
| C/N | -0.94 | 0.82 | 0.37 | -0.17 |
| P | 0.56 | 0.35 | 0.81 | -0.91 |
| K | **1.00** | -0.53 | 0.04 | -0.24 |
| Mg | 0.41 | 0.50 | 0.90 | **-0.97** |
| Fe | **0.99** | -0.50 | 0.07 | -0.27 |
| Ca | 0.72 | 0.14 | 0.67 | -0.81 |
| S-SO_4_ | **-0.98** | 0.75 | 0.25 | -0.04 |
| B | 0.71 | 0.15 | 0.68 | -0.81 |
| Mn | **1.00** | -0.53 | 0.04 | -0.24 |
| Cu | -0.86 | 0.09 | -0.48 | 0.65 |
| Zn | -0.96 | 0.33 | -0.25 | 0.45 |
| Al | 0.67 | 0.21 | 0.72 | -0.85 |
| Na | 0.01 | -0.82 | **-1.00** | **0.98** |

in bold – statistical significant values (P≤0.05); in red – values suggesting potential toxicity (discussed in the text)

**Table S5.** Relationships between the physico-chemical properties and ecotoxicological parameters in the soil with 20% dose of mining waste (MS)

| Properties | V. fisherii (Microtox®) | *Daphnia magna* 24h (Daphtoxkit F) | *Daphnia magna* 48h (Daphtoxkit F) | *Lepidium sativum* (Phytotoxkit F) |
| --- | --- | --- | --- | --- |
| pH | 0.13 | -0.87 | **-1.00** | 0.09 |
| C | -0.50 | **-0.99** | -0.79 | -0.53 |
| N | -0.08 | -0.95 | **-0.98** | -0.11 |
| C/N | -0.51 | 0.60 | 0.92 | -0.48 |
| P | -0.61 | **-0.97** | -0.71 | -0.64 |
| K | 0.13 | -0.87 | **-1.00** | 0.09 |
| Mg | -0.34 | 0.74 | **0.98** | -0.30 |
| Fe | 0.13 | -0.87 | **-1.00** | 0.09 |
| Ca | -0.10 | 0.88 | **1.00** | -0.07 |
| S-SO_4_ | -0.17 | 0.85 | **1.00** | -0.13 |
| B | -0.93 | -0.69 | -0.24 | -0.94 |
| Mn | 0.13 | -0.87 | **-1.00** | 0.09 |
| Cu | -0.09 | 0.89 | **1.00** | -0.05 |
| Zn | -0.03 | 0.91 | **0.99** | 0.01 |
| Al | -0.37 | 0.71 | **0.97** | -0.34 |
| Na | **1.00** | 0.42 | -0.09 | **1.00** |

in bold – statistical significant values (P≤0.05)
